# Emerging memristive neurons for neuromorphic computing and sensing

**DOI:** 10.1080/14686996.2023.2188878

**Published:** 2023-04-19

**Authors:** Zhiyuan Li, Wei Tang, Beining Zhang, Rui Yang, Xiangshui Miao

**Affiliations:** a School of Integrated Circuits, School of Optical and Electronic Information, Huazhong University of Science and Technology, Wuhan, China; b Hubei Yangtze Memory Laboratories, Wuhan, China

**Keywords:** Memristive devices, artificial neurons, spiking dynamics, neuromorphic computing, neuromorphic sensing

## Abstract

Inspired by the principles of the biological nervous system, neuromorphic engineering has brought a promising alternative approach to intelligence computing with high energy efficiency and low consumption. As pivotal components of neuromorphic system, artificial spiking neurons are powerful information processing units and can achieve highly complex nonlinear computations. By leveraging the switching dynamic characteristics of memristive device, memristive neurons show rich spiking behaviors with simple circuit. This report reviews the memristive neurons and their applications in neuromorphic sensing and computing systems. The switching mechanisms that endow memristive devices with rich dynamics and nonlinearity are highlighted, and subsequently various nonlinear spiking neuron behaviors emulated in these memristive devices are reviewed. Then, recent development is introduced on neuromorphic system with memristive neurons for sensing and computing. Finally, we discuss challenges and outlooks of the memristive neurons toward high-performance neuromorphic hardware systems and provide an insightful perspective for the development of interactive neuromorphic electronic systems.

## GRAPHICAL ABSTRACT

**Figure:**
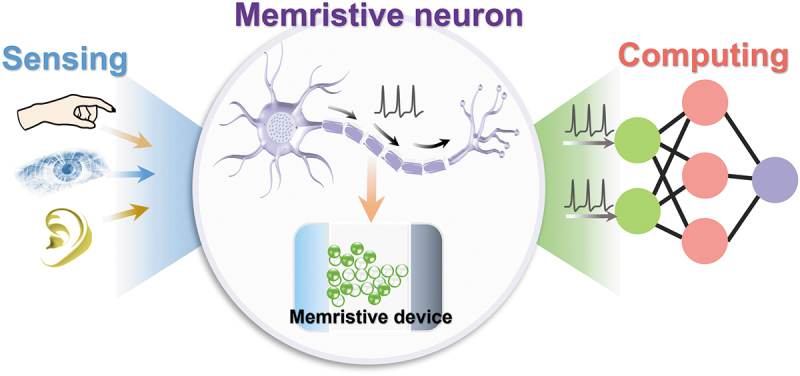

## 1. Introduction

Over the past few decades, significant progress has been achieved in artificial intelligence (AI) as a result of availability of big data, increased computational power, and development of machine learning, with even more dramatic changes envisioned in the further [1–4]. However, the rapid development of AI technology poses considerable challenges for the underlying electronic hardware and system, particularly their energy consumption [5]. Traditional mainstream hardware platforms are based on von Neumann architecture in which computing and storage units are physically separated, so computing requires continuously swapping of data between the units. This architecture is efficient for precision computing tasks, but becomes inefficient when it comes to handling unstructured data-intensive applications required for AI. Thus, it is urgent to develop new computing architectures and devices.

Actually, in 1990 Carver Mead first proposed to build neuromorphic (bio-inspired) computing systems to overcome the bottleneck of von Neumann architecture, and even to rival or exceed the cognitive capabilities and energy efficiency of human brain via electronics [6]. That is because human brain is a massively complex 3D neural network via petascale parallel architecture for information processing and communication with ultra-low power consumption (~20 W) [7,8]. Moreover, inspired by the biological perceptual system, researchers have devoted much more efforts to develop the human-machine interfaces in few years. In the interfaces, the neuromorphic systems with perception functions are of great significance to handle complex environmental information [9–12]. However, traditional perception system involves a separate process of detecting (analog sensing signals) and converting (digital computing signals) information obtained from the physical world using diverse sensors and analog-to-digital converters (ADCs). As the number of sensor nodes increases (40 billion by 2022), this architecture leads to a large amount of redundant data being exchanged between sensory terminals and computing units, which seriously deteriorates the latency of decision-making and inevitably increases the overall computing energy consumption [13,14]. Thus, how to design interactive neuromorphic systems to integrate sensing, processing, computing, and decision-making capabilities with energy-efficient and high processing speed, is highly desirable for developing a next-generation AI systems paradigm.

However, many neuromorphic chips, such as TrueNorth [15,16], SpiNNaker [17], and Loihi [18,19], have relied heavily on traditional silicon-based complementary metal-oxide-semiconductor (CMOS) technology. In detail, electronic synapses and neurons, which require complex digital/analog CMOS circuits to maintain biological plausibility, serve as building blocks for artificial neural networks (ANNs). These CMOS synapses and neurons are inefficient in terms of the chip area and energy consumption. Meanwhile, CMOS technology is reaching its physical limits, so further downscaling and integration of the processors is becoming difficult [20,21]. Fortunately, the memristive device has emerged as a novel neuromorphic device in the last decades [22–28]. The memristive device can work as a core component (i.e. synapses and spiking neurons) of neuromorphic systems, benefiting from their excellent advantages, such as ultrafast switching speed (~ns scale), high endurance (>10^12^ cycles), high integration, low power consumption (~fJ range), and promising bio-plausibility. Specifically, memristive sensory neurons provide the neural sensing and processing functions with human-like abilities [29–31]. These sensory neurons can convert the external sensing signals into electrical signals without ADCs module, resulting in high energy efficiency and low latency. Considering the prospect that the memristive device brings to bio-inspired computing systems, it is urgent to review recent development of artificial spiking neurons and sensory neurons at the device and system levels.

In recent years, many valuable reviews on emerging memristive devices from the perspectives of memristive materials [32,33], switching mechanisms [34,35], and neuromorphic systems [36–39] have been published. For example, Bian et al. highlighted memristive materials and strategies that implement synaptic functions for neuromorphic computing [38], while Yang et al. provided a comprehensive overview on the development of neuromorphic engineering from biological nervous systems to spike-based neuromorphic computing systems [39]. Different from the existing literatures, we present the first comprehensive overview of recent advances in the development of neuromorphic perception and computing systems from the perspectives of memristive neurons, aiming to give guidelines for future research toward development of bio-inspired computing and robotics. This review is organized as follows: we first provide an overview of biological neurons and various neuron models. Subsequently, we introduce the switching mechanisms of memristive devices, and discuss how to realize neuron based on these mechanisms. Next, we focus on the spiking behaviors that are implemented utilizing the simple circuit and inherent switching dynamic processes of memristive devices. Then, at the system level, some remarkable progress of memristive neurons for bio-inspired computing and sensing are presented. Finally, we shed light upon the remaining challenges and suggest outlooks on the development of the future interactive neuromorphic system.

## 2. Biological neurons

To better understand biological neurons, we first need to know the biological nervous system in which neurons works as the essential computing units. As well known, the nervous system of human being is the most complex information processing system that has evolved over millions of years [40]. It possesses approximately 86 billion neurons, each of which can form up to ~10,000 connections with other neurons through synapses, 99.9% of which are distributed in the brain [40,41]. Thanks to this tremendous neural network with strong robustness, superior error-tolerance, highly energy-efficient manners, and extremely low power consumption (1 ~ 10 fJ per event), human can handle high-level cognitive function, such as information integration, recognition, reasoning, imagination, etc [42,43]. In the nervous systems, biological neurons serve as the core computing unit. They are mainly nonlinear information processing and transmission units, that generate action potentials when receiving excitatory or inhibitory stimuli, and then transmit them to the next neurons. The action potential with different patterns encodes the information transmitted in the network. In the following sections, we conduct a discussion on biological neurons in terms of spiking neuronal properties (Section 2.1) and its models (Section 2.2).

### 2.1. Biological neuron properties

**Figure 1.**
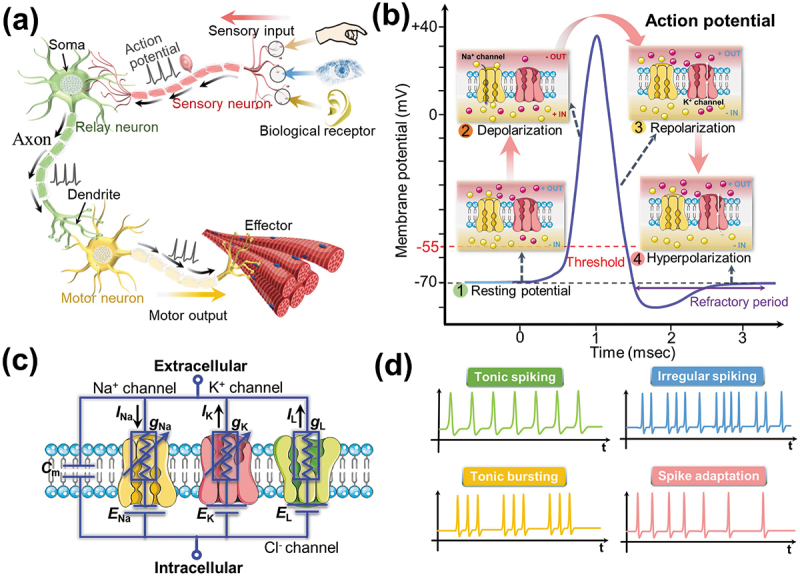
(a) Schematic of a biological nerve circuity including three different neurons: sensory neuron, relay neuron and motor neuron. (b) An action potential with ion exchange processes of Na^+^ and K^+^ channels. (c) Hodgkin-Huxley neuron model. (d) Examples of the biological spiking behaviors, including tonic spiking, tonic bursting, irregular spiking, and spike adaptation.

Neurons, also known as nerve cells, are living electrochemical systems that are separated internally and externally by a neuronal membrane. In biological neural systems, there are a great variety of neurons with different structures and functions. For example, Figure 1(a) shows that a simple nerve circuity includes three different neurons: sensory neurons, relay neurons and motor neurons. Sensory neurons, also known as afferent neurons, are connected to a sensor (e.g. touch, vision, hearing, smell, etc.); motor neurons, also referred to as efferent neurons, are connected to muscle fibers (govern movement); while relay neurons, also referred to as interneuron, connect various neurons (e.g. sensory and motor neurons) within the brain and spinal cord, and are easy to recognize, due to their short axons. Information related to sensory-cognition-motor is transmitted between these different types of neurons that supervise the conveyance of information related to sensory-cognition-motor.

Despite this great variety, they all have a typical neuronal structure, comprising of three main parts: (i) Dendrites, which are the tree-root-shaped part of the neuron which are usually extending from the soma. Dendrites receive neural signals from pre-synaptic neurons and then transmit them to soma. It has been found that many complex calculations can be done in dendrites before the signal reaches soma, including Boolean operating, coincidence detecting, etc [44,45]. (ii) The soma, also called cell body, which is the essential center of the neuron. The soma synthetizes neurotransmitters, generates an action potential and then sends it to the axon. (iii) The axon, also referred to as nerve fiber, is a tail-like structure of the neuron which joins the soma at a junction called the axon hillock, acting as the output channel of neuron signals in biological system. The function of the axon is to transmit neuron signals away from the soma to the post-synaptic neurons.

The working process of biological neurons involves complex ion dynamics processes. The neuronal membrane serves as a barrier between the external environment and the neuronal cytoplasm, across which various ion exchange processes take place. These processes are in turn governed by voltage-dependent opening and closing of ion channels (e.g. Na^+^ and K^+^ ion channels) [46,47]. As illustrated in Figure 1(b), an action potential can normally be roughly divided into four segments: resting potential, depolarization, repolarization, and hyperpolarization. Initially, the neuron is in a resting potential (usually ~−70 mV), the membrane potential maintains a constant charge gradient (Na^+^/K^+^ pump). When incoming spikes induce the membrane potential to reach the threshold of the neuron (usually ~−55 mV), Na^+^ ion channels are activated, and the rapid influx of Na^+^ ions results in depolarization of the membrane potential. Then, the voltage-gated K^+^ ions channels determine physiological processes of repolarization and hyperpolarization. Ion pumps allow K^+^ ions to flow out of the cell membrane, the membrane potential decreases rapidly, until it reaches a new resting state when the outward of K^+^ ions balance the inward of Na^+^ ions. This spike generation process is an all-or-none event, a spike generates when its membrane potential exceeds the threshold; Otherwise, the membrane potential boosting lasts for a short time without leading to spike generation. Note that after the neuron emitting an action potential, it remains nonresponsive to subsequent stimuli for a certain period of time, called as the refractory period.

### 2.2. Biological neuron models

With the rapid development of neuroscience, artificial neuron models have made a great contribution to the development of neuronal dynamics [48]. Having known the generic biological nature of neuronal functionality, it is obvious that a resulting model, describing a biological neuron, would consist of complex dynamical equations. In 1952, Hodgkin and Huxley proposed a detailed mathematical model, that is, the Hodgkin-Huxley (HH) model, which offers a great deal of fidelity to the biologically neuronal dynamics [49]. This biologically plausible neuron model simulates the dynamic relationship between the membrane potential and the ionic current flow across the neuronal cell membrane. As shown in Figure 1(c), the HH model regards the cell membrane components as an equivalent circuit, which describes the dynamic change between the ionic current flow across membrane potentials versus time for a spike. The phospholipid bilayer in the cell membrane is represented by capacitance (*C*_m_). The variable conductance (*g*_Na_, *g*_K_) and linear conductance (*g*_L_) serve as the Na^+^, K^+^ and Cl^-^ ion channels, respectively; where the *I*_Na_ and *I*_K_ represent the currents through the corresponding Na^+^ and K^+^ ion channels, while *I*_L_ is responsible for introducing leakage channel currents. In addition, electrochemical gradients are represented by voltage sources (*E*_ions_). Benefiting from multiple parameters and complex variable relationships, the HH model can well describe many neuronal spiking patterns, including integrate-and-fire, refractory period, tonic spiking, phasic spiking, tonic bursting, phasic bursting, irregular spiking, spike frequency adaptation, etc. Neuronal spiking behaviors are partially demonstrated in Figure 1(d). These diverse spiking behaviors are of significant importance to develop versatile, adaptive, robust, and general-purpose brain-like computing systems [50–52]. To reduce the mathematical complexity of the model, a variety of simplified versions of the HH model (i.e. Morris-Lecar model, Izhikevich model) were proposed, which also implements adequately neuronal dynamics to the neural network [53–55]. In contrast to the HH model, it is difficult to achieve a complete pattern of biological neuronal spiking.

Although the HH, Morris-Lecar model and Izhikevich model are biophysically meaningful and measurable, their application to ANNs is limited by the difficulties posed to the analysis of dynamical systems with high-dimensional nonlinear equations [56,57]. Hence, a group of highly simpler biologically realistic neuron models had been proposed. In 1907, Lapicque proposed the integrate-and-fire (IF) neuron model to implement the basic functionalities of biological neurons [58]. Nevertheless, this neuron model does not account for the specific shape of an action potential and lacks of rich spiking behaviors, which differ greatly from biological neuron. In principle, a localized graded membrane potential should show short-term dynamic behavior, leading to subthreshold membrane boost that leaks out rapidly. Thus, a complementary leaky term is added to the membrane potential to form the leaky integrate-and-fire (LIF) neuron model, which endows the neuron with biologically plausibility [59]. The LIF neuron model, based on simpler linear equations with a single variable, has rendered them the most popular models in computational neuroscience. For example, owing to its leaky nature, this model is commonly used for the domain of spiking neural networks (SNNs) to render a regularizing effect on their firing rates [60]. In addition, various derived IF models have been developed to implement more abundant neuronal dynamic behaviors, such as spiking frequency adaptation, tonic bursting and spiking latency, etc [61–64].

## 3. Memristive mechanisms for artificial neurons

**Figure 2.**
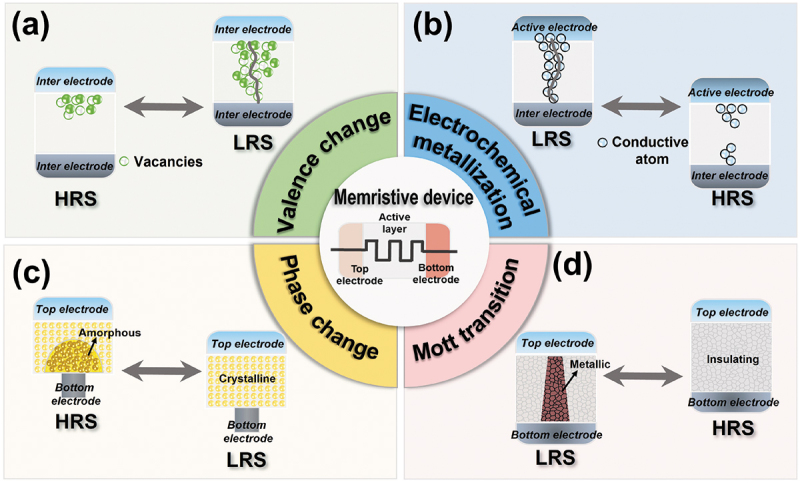
Schematic of various switching mechanisms underlying memristive devices for neuron demonstration. (a) Valence change. (b) Electrochemical metallization. (c) Phase change. (d) Mott transition.

With their unique advantages, emerging memristive devices have provided an alternative approach in recent years for building advanced AI systems [65]. The mechanisms of memristive devices are very complicated due to the coupled thermal, chemical, and electrical dynamics processes. Generally speaking, the mechanisms can be divided into valence change mechanism (VCM), electrochemical metallization mechanism (ECM), phase change mechanism (PCM), and Mott transition mechanism, as shown in Figure 2. These mechanisms endow the memristive devices with abundant nonlinear dynamics and can provide us opportunities for constructing biologically plausible neuron. In subsequent sections, devices based on these mechanisms and their principles for constructing memristive neurons are discussed.

### 3.1. Valence change for memristive neurons

A typical VCM device usually adopts a simple sandwich structure with inter-electrode/active layer/inert electrode, as depicted in Figure 2(a). Inert metals such as Pt [66], Ta [67], TiN [68], and Pd [69] were often used as the electrode materials. VCM generally occurs in transition metal oxides (e.g. TiO_x_ [70], TaO_x_ [71], WO_x_ [26], etc.) of the active layer, in which anions, like oxygen ions (oxygen vacancies), migrate under an electric field (E-field) and thermal effect, resulting in a relatively high mobility. The resistive switching is dominated by a localized filament region, and SET and RESET processes are achieved through the formation and dissolution of oxygen-deficient filaments based on oxygen ion/vacancy transport. More specifically, for SET, an applied electric field induces a soft breakdown in the oxide and creates a local conductive pathway consisting of oxygen vacancies, inducing a transition from the high-resistance state (HRS) to low-resistance state (LRS). For RESET, the filament is ruptured either by a recombination of oxygen vacancies with oxygen ions under an applied electric field with opposite polarity or through Joule heat under a larger electric field (unipolar as polarity of the E-field does not matter), inducing LRS back to HRS. The charged ions and vacancies of the switching layer provide an underlying force for VCM artificial neuron. However, most VCM memristors exhibit the common non-volatile switching behavior, which can only be fulfilled the summator function of biological neurons [72,73]. The additional circuits to implement the evaluation of the threshold membrane potential, spike generator, and feedback path to reset the device to its primary state after firing. This scheme further leads to greater chip area overhead and greater energy consumption in the actual chip application. Moreover, it will increase the complexity of the operation, as each new input must wait for the completion of the previous reset process, especially in systems with rate coding schemes.

### 3.2. Electrochemical metallization for memristive neurons

Similar to VCM devices, ECM resistive switching still relies on the conductive filament. The ECM memristor also has a sandwich structure (Figure 2(b)). However, their mechanism is mainly attributed to the redox reaction of the active metal electrode, such as Ag [74], Cu [75], Ni [76], etc. The switching layer usually uses diverse solid electrolyte materials such as Ag_2_S [77], Cu_2_S [78], SiO_2_ [79], SiO_x_:Ag [80], etc. The dynamics of metal nanoparticles contained in the active layer play a significant role in implementing ECM memristors [74,81]. Metal cations from the active electrode migrate toward the inter electrode under an external E-field, and are gradually formed a conductive filament in the conducting layer, leading to an abrupt conductance increase of the device. The detailed process is as follows. For SET, active metal atoms are oxidized into cations that are injected into the switching layer, move under the applied E-field, and eventually form a metallic pathway that bridges to the other electrode, resulting in the device switching from HRS to LRS. For RESET, the conductive pathway is disrupted by an E-field with opposite polarity, leading to the device back to HRS. It should be noted that when the conductive filaments are weak, once the external E-filed is removed, the filament will break spontaneously and form a series of nanoparticles through diffusion, due to the result of Joule heat and interfacial energy [77,82]. The volatile ECM device (diffusive memristor) at this point has volatile TS behavior. The formation and rupture of conductive filaments based on charged metal ions serve as a principal mechanism for the ECM artificial neuron [79,83,84]. In ECM neuron, a rapid increase in the conductance state can mimic the IF characteristics of neurons; a spontaneous relaxation process in the conductive pathway is similar to the leaky property of the membrane potential in a biological neuron. Therefore, a volatile ECM device has inherent neuron dynamic characteristics, which can facilitate the design of more compact and simplified neuron circuits.

### 3.3. Phase change for memristive neurons

As shown in Figure 2(c), a typical PCM cell consists of a phase change material sandwiched between two metallic electrodes, with a mushroom structure. The family of chalcogenides (Ge, Sb, and Te alloys) has been widely and deeply studied as phase change materials [85–87]. PCM can be switched between the amorphous states with low electrical conductivity and the crystalline states with high electrical conductivity, corresponding to the HRS and LRS, respectively. The reversible change of phase change material between HRS and LRS is driven by Joule heating. Thus, a ‘heater’ is important in PCM cell to generate enough heat to induce the phase transition when an extrinsic voltage pulse is applied [88]. For SET process, a low yet wide voltage pulse is applied and generates a sudden increase in electrical conductivity in amorphous region. Subsequently, heats up the cell to gradual crystallization with a local temperature in the range of 500–700 K, switching the cell from HRS to LRS. While in RESET, a short yet high voltage pulse is needed to heat up a significant part of the device to above material melting temperature (~1000 K). Then, device back to the HRS through the rapid quenching step to freeze the molten material. The SET transition process takes long latency but low power since the crystallization process is related to atomic movement; whereas the reset transition takes short latency but high power as the device needs to be heat up to its melting temperature. The reversible amorphous-to-crystalline phase transition serves as a principal switching mechanism for PCM artificial neuron [87,89]. Threshold switching for the neuronal firing dynamics is expected in this PCM device, because of the continuous growth of the crystalline phase under an E-field. However, similar to nonvolatile VCM neuron, one of the potential drawbacks of PCM neurons is the nonvolatile nature of the device, which requires additional feedback circuits to reset devices to their primary state after every firing process [90,91].

### 3.4. Mott transition for memristive neurons

Two-terminal Mott devices can also be referred to as another class of phase change device in a broad sense. Unlike the PCM devices, the Mott transition occurs between metallic and insulating states (Figure 2(d)). This device usually exhibits current-controlled negative differential resistance (NDR) switching effect, which is a TS behavior, caused by reversible thermally driven metal-to-insulator phase transition (MIT) of the Mott materials (e.g. NbO_x_, VO_2_,) [92,93]. The phase transition (also called Mott transition) can be triggered by a variety of methods including temperature, stress, doping, electrical, optical and magnetic stimuli [94,95]. At the level of device, the voltage triggered Mott memristor has been extensively explored in recent years, because the bias control makes the conductance programming more convenient. In the case of the low valence vanadium oxide (e.g. VO_2_ and V_2_O_3_), they exhibit satisfactory transition temperature (*T*_c_) that is slightly higher than the room temperature [96,97]. Therefore, the phase transition of the VO_2_-based memristor from a monoclinic insulator to a rutile metal phase is easily accessible. A dramatic transition from a monoclinic insulator (HRS) to rutile metal phase (LRS) is observed when a voltage pulse exceeding the threshold is applied. After the voltage pulse is terminated, the LRS spontaneously relaxed into the HRS. Similar to ECM neurons, this volatile TS behavior in voltage-controlled mode has attracted much attention for the potential in building artificial neuron [98–100]. Moreover, through elaborately engineering the device structure, the abundant intrinsic switching dynamics could be observed in the higher-order Mott memristor, leading to rich neuronal dynamic behaviors [101,102]. This approach is described in Section 4.2.

## 4. Memristive neurons for spiking behaviors

Inspired by the working mechanism of the human brain, artificial neuronal spiking dynamic behaviors play an indispensable role in information communication and processing of implementing bio-inspired computing systems. Traditionally, researchers were dedicated to designing for various analog/digital CMOS circuit architectures to realize required functions of the artificial neurons [103–106]. Despite these implements can provide a high accuracy for neuronal dynamics emulations, they also suffer from high energy consumption and large silicon area. The unique nonlinear electrical characteristics and internal dynamics in the above (Section 3) emerging memristive devices provide a novel substrate for mimicking neuronal dynamic behaviors. In Section 4, we discuss implementations of spiking neurons in memristive devices for emulating different spiking dynamic behaviors.

### 4.1. Memristive neurons for simple spiking behaviors

**Figure 3.**
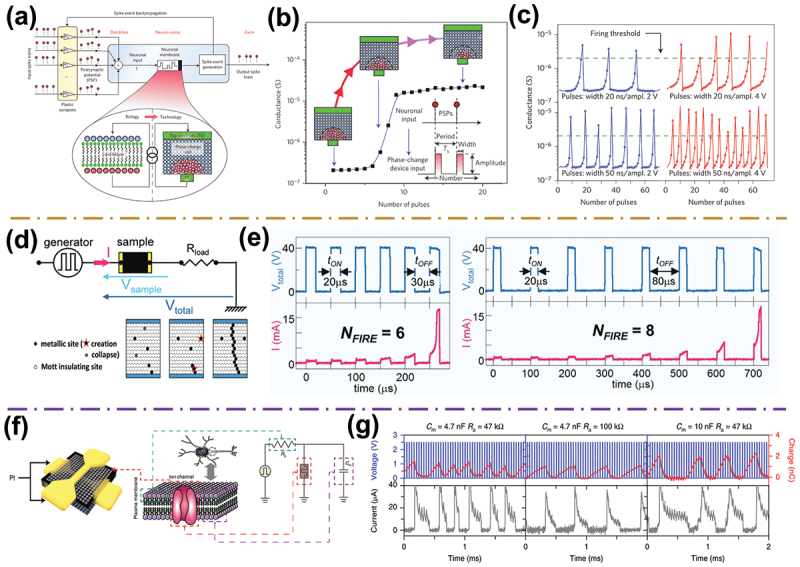
Bioplausible memristive neurons with simple spiking dynamic behaviors. (a) Schematic of artificial neuron based on a phase-change device with a plastic synaptic input array. (b) The conductance changes of a phase-change cell when applying a series of crystallizing pulses. (c) The if dynamics in the PCM neuron. Reprinted with permission from [91]. Copyright 2016 Springer Nature. (d) Schematic diagram of the LIF model implementation with a GeTa_4_Se_8_-based mott memristor and a resistor; the LIF functions thanks to the accumulation of correlated metallic sites. (e) Experimental neuron spiking firing behavior obtained by applying trains of short pulses with various t_ON_ and t_OFF_. Reprinted with permission from [107]. Copyright 2017 WILEY-VCH. (f) Schematic illustration of the artificial LIF neuron circuit with a SiO_x_N_y_:Ag-based diffusive memristor. (g) Controlled firing response of LIF neuron under different circuit parameters (C_m_ and R_a_). Reprinted with permission from [83]. Copyright 2018 Springer Nature.

The indispensable function of a spiking neuron is the integration and threshold firing of membrane potentials during the communication of the nervous system. IF models are mainly used to realize this simple neuronal function without the decay of membrane potential over time. This model is the simplest of all neuron models that lower computational requirements are required compared to other models, thus the hardware cost is relatively low. One novel strategy to build IF model is to employ emerging memristive devices that are high scaling, high efficiency, and abundant internal dynamics. The IF behavior is emulated by a strong nonlinear transition in memristors, like the abrupt increase in conductance in memristive devices. For example, Tuma et al. implemented an IF functionality by using a chalcogenide (GST)-based PCM device [91]. As shown in Figure 3(a), the core idea of the PCM neuron is to represent the neuronal membrane potential through the phase configuration within the nanoscale phase-change device. The evolution of the membrane potential is driven directly by the reversible amorphous-to-crystalline phase transition dynamics in the PCM device and is altered over time by applying short electrical pulses based on the neuronal input. In this device, the phase-change memory process depends on the number of crystallizing pulses. After a certain number of voltage pulses (approximately six pulses), the conductance of the device rises sharply, causing the neuron to cross the firing threshold (Figure 3(b)). Then, a reset voltage pulse is applied to device to reset the neuronal potential. The neuronal spiking rate can also be controlled by the width and amplitude of the crystallizing pulse (Figure 3(c)). After the PCM neuron is reset, the thickness of the amorphous region produced by the melt-quench process and its internal atomic configuration are different from the previous state. Hence, the spiking behavior of the neuron presents an approximately normal distribution of the spike intervals (i.e. inherently stochastic) under multiple IF cycles. This neuronal stochastic spiking behavior is pivotal for population-based neuronal computations [108].

In computational neuroscience, the LIF neuron model has been developed to enhance the model’s bioplausible, after combining the IF neuron with leakage of membrane potential. Currently, LIF models are more prevalent in bio-inspired computing system with computational efficiency [109,110]. In practical hardware implementations, how to achieve the ‘leaky’ function is especially a challenge. Encouragingly, the leaky feature is enabled by the spontaneous relaxation in the conductive path (i.e. volatile properties) in the memristive devices, including heat dissipation in MIT devices [100,107], ion diffusion in the ECM or VCM devices [68,79,111,112], spontaneous depolarization in ferroelectric devices [113,114], as well as tunable magnetization in spintronic devices [115,116]. For example, Stoliar et al. proposed a simple two-terminal GeTa_4_Se_8_-based MIT device, which could be well implemented for LIF spiking behavior [107]. As illustrated in Figure 3(d), the whole LIF model module was implemented by a Mott memristor and a resistor. A dramatic transition from a low to high conductance state (that is, insulating state to metallic state) was observed in the GeTa_4_Se_8_ insulator with a narrow gap when a voltage pulse exceeding the threshold was applied (Figure 3(d)). After the voltage pulse was terminated, the HRS spontaneously relaxed into the LRS. These two features enabled the implementation of a LIF neuron function in the device. Spiking responses of the current (i.e. firing) and spontaneous relaxation (i.e. leaky) were demonstrated using pulse trains with various widths (t_ON_) and periods (t_OFF_), as shown in Figure 3(e). Kurenkov et al. reported an artificial LIF neuron based on spin-orbit torque (SOT) device [116]. The dynamics of SOT switching in antiferromagnet/ferromagnet heterostructures was used to implement LIF neuronal functionality. The two magnetization states were associated with a nonfiring and firing states of a neuron.

Another appealing solution to realize the LIF neuron is the use of the volatile TS memristor wired with a series resistor and a parallel capacitor. For example, Wang et al. reported a LIF neuron by combining a SiO_x_N_y_:Ag-based diffusive memristor (functions as an ion channel), an axial resistor (R_a_) and a parallel membrane capacitor (C_m_) (Figure 3(f)) [83]. The memristive ECM neuron emulated some neuronal functionalities, including stochastic leaky integrate-and-fire and strength-modulated frequency response. As shown in Figure 3(g), with one parameter fixed, a smaller R_a_ results in high-frequency spiking, whereas a larger C_m_ leads to low-frequency spiking. The flexibility and applicability of the ECM neuron is conducive to achieving desirable spiking dynamics characteristics for specific applications. In some memristive devices with a volatile effect but no obvious threshold switching value, LIF neuron can also be implemented. Recently, Park et al. developed an artificial LIF neuron based on gradual TiO_x_-based VCM memristor without TS characteristic [117]. With the self-diffusion of oxygen anions in a thin gradual TiO_x_ layer, which resulted in less stable and highly volatile switching behaviors and was utilized as the neuronal dynamics. The artificial neurons have high spatiotemporal uniformity and have been used to construct neuromorphic hardware capable of processing sequential data.

### 4.2. Memristive neurons for complex spiking behaviors

**Figure 4.**
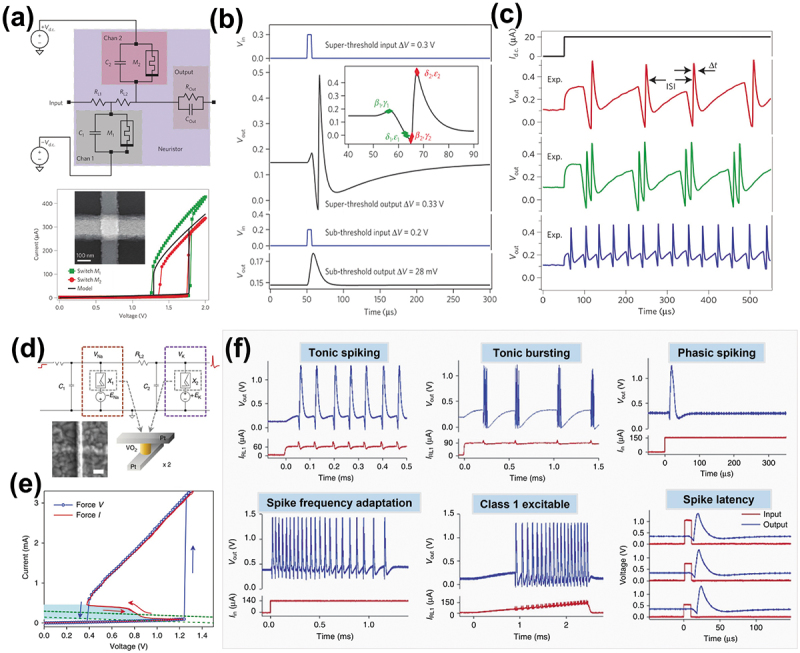
Biophysical memristive neuron with complex spiking dynamics. (a) HH neuristor circuit diagram and I-V characteristics of NbO_x_-based mott memristor. (b) All-or-nothing neuron spiking behavior of the neuristor. (c) Experimental spike outputs of the memristive HH neuron with different circuit parameters. Reprinted with permission from [119]. Copyright 2013 Springer Nature. (d) Implementation of a HH neuron with two VO_2_-based mott memristors. (e) Typical voltage-controlled (force V) and current-controlled (force I) I-V characteristics of a VO_2_-based mott memristor. (f) Partial schematics of the 23 biological neuron spiking behaviors experimentally demonstrated by memristive HH neuron circuit, including tonic spiking, tonic bursting, phasic spiking, spike frequency adaptation, class 1 excitable, and spike latency. Reprinted with permission from [120]. Copyright 2018 Springer Nature.

The above-mentioned artificial memristive neurons have realized the basic neuronal functions (i.e. IF or LIF) and have not demonstrated more rich neuronal dynamics and computational complexity of biological neurons. Notably, neuronal functions are often higher in complexity to efficiently perform temporal processing of information [118]. Thus, research had proposed a detailed biologically neuron model, that is, HH model. Unlike IF or LIF neurons that can be realized in neuron circuit based on a single memristive device, the HH neuron, involving complex neuronal dynamics of Na^+^ and K^+^ ion channels, requires more complex memristor circuits, and the progress on realization of artificial HH neuron is quite limited. For example, Williams et al. firstly proposed a neuristor built using two NbO_x_-based Mott memristors [119]. As illustrated in Figure 4(a), the neuristor circuit comprises two channels, each of which uses a Mott memristors (M_1_ or M_2_) and a parallel membrane capacitor (C_1_ or C_2_) to function as Na^+^ and K^+^ ion channels of the HH model. The two channels are energized with DC voltage (V_dc_) of opposite polarity and coupled to each other through a load resistor (R_L2_). The core memristive device exhibits the volatile TS behavior arising from an insulator-to-metal phase transition induced by Joule heating. Figure 4(b) demonstrates the all-or-nothing spiking behavior of the neuristor induced by a super-threshold pulse. Moreover, the neuristor can be easily modified to provide other biomimetic spiking patterns essential for action-potential-based computing: regular spiking, chattering, and fast spiking (Figure 4(c)).

To further obtain the diversity of spiking behaviors, Yi et al. experimentally demonstrated biologically plausible and stochastic HH neuron built with VO_2_-based Mott memristive devices in a more concise circuit [120]. Similarly, the voltage-gated Na^+^ or K^+^ ions channel is also emulated by DC voltage (−E_Na_ or +E_k_) of opposite polarity and a Mott memristor, which is closely coupled with a parallel membrane capacitor (C_1_ or C_2_) and a series load resistor (R_L1_ or R_L2_), as shown in Figure 4(d). Figure 4(e) exhibits a wide hysteresis loop (TS behavior) in the voltage-controlled mode, and a much narrower hysteresis (hysteretic negative differential resistance) in certain region of the I-V curve, all due to the Mott transitions. According to the different input sources, excellent MIT process of the Mott memristors, and the change of parallel capacitors, the system produced 23 different biologically plausible neuronal behaviors, such as tonic spiking and bursting, spike frequency adaptation, spike latency, and so on (Figure 4(f)). Moreover, this work provides a possible fabrication procedure for stackable integrated memristive HH neuron. The integrated neuron only requires up to three layers of interconnect metals, however, such a connectivity cannot be easily achieved using conventional CMOS technology [121]. Thus, it is essential that the implementation complexity of HH neuron circuit be simplified to realize rich spiking dynamics.

**Figure 5.**
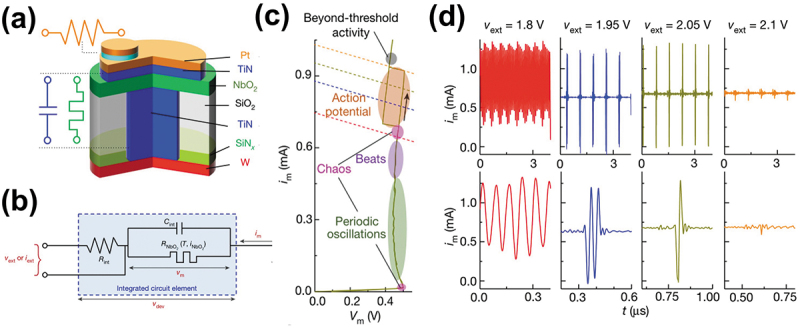
(a) Structure diagram of third-order NbO_2_-based mott memristor. (b) The equivalent circuit model of the third-order nanocircuit element. (c) Quasistatic I-V behavior of third-order NbO_2_-based device. (d) Measured various neuron spiking dynamic behaviors of the third-order NbO_2_-based device with different applied external voltages. Reprinted with permission from [102]. Copyright 2020 Springer Nature.

Innovatively, Kumar et al. reported a third-order nanocircuit element (third-order memristor) based on NbO_2_ Mott transition dynamics to perform many key neuronal spiking behaviors [102]. The proposed third-order memristive neuron, which consists of a NbO_2_-based Mott volatile memristive switch, an internal series resistor defined by an electrode interface), and an internal parallel capacitor defined by the metal contacts clamped in the dielectric (Figure 5(a)). Note that the device includes three state variables: temperature, charge on internal capacitor and speed of Mott transition, each of which corresponds to a dynamic physical process. In order to induce the desired higher-order Mott transition dynamics, the geometric structure and material stoichiometry of the neuron devices were carefully designed to emulate rich neuronal dynamics. The equivalent circuit model of the device is shown in Figure 5(b). When powered by a tunable constant bias voltage, different regions of its I-V curve are accessed by load lines determined by the internal resistor and the applied voltage (Figure 5(c)). Interestingly, the single neuron device could produce 15 different neuronal dynamics by tunning the voltage across the device, such as spiking, bursting, and chaos, etc., as shown in Figure 5(d). This novel work provided new insights into very compact and densely functional biologically plausible neuromorphic computing.

## 5. Memristive neurons for neuromorphic computing

**Figure 6.**
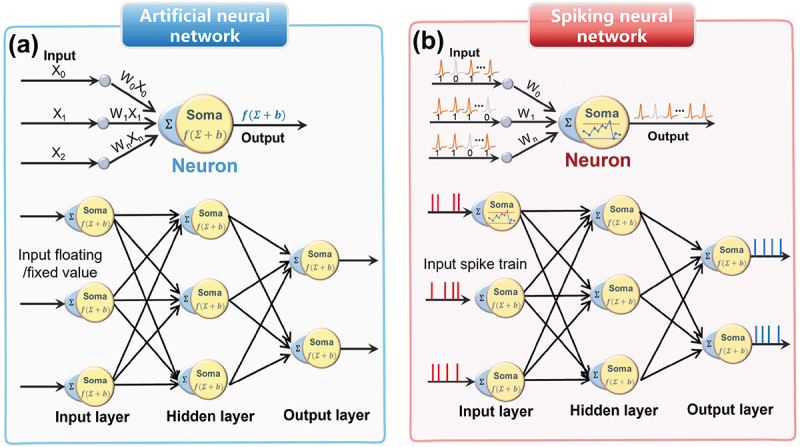
Comparisons of ANN and SNN. (a) Illustration of non-spiking ANN, where neuron is determined by the activation function to process numerical value input. (b) Illustration of SNN, where neuron is determined by the biological spiking dynamics to process the event-based spike input.

In the era of AI, neuromorphic computing, a parallel computing architecture, has become a promising candidate for high-performance hardware systems. Scientists proposed artificial neurobiological network models (i.e. SNNs and ANNs) to develop neuromorphic computing, as illustrated in Figure 6. ANNs can perform supervised, semi-supervised, unsupervised, and reinforcement learning algorithms, and excel in deep learning tasks that had large amounts of computational resources for the training data [2,122]. They usually receive consecutive values and output consecutive values (i.e. floating point, fixed point or analog value), biologically inaccurate and do not mimic the abundant dynamics of biological spiking neurons. In contrast, SNNs, as third generation neural network, mimic the brain processes information more faithfully, in which the internal neurons communicate with each other through the sequence and timing binary spiking signals (i.e. a rate-coding or spatiotemporal-coding) [60,123,124]. When processing complex temporal intelligence tasks (e.g. event-driven information processing), SNNs can show great advantages over ANNs. However, traditional SNN-based hardware implementations usually were implemented by CMOS circuits and external waveform generators, which require more complex circuit design, higher power consumption, and larger chip space [16,52,125]. The emerging memristive devices provide new insights into highly efficient and compact SNNs hardware system. The nonlinearity and dynamics in the above artificial memristive neuron provide vital substrates for implementation of SNNs. In this section, we conduct a comprehensive review of building a spike-based neuromorphic hardware system exploiting memristive neuron.

### 5.1. Memristive LIF neurons for SNN computing

**Figure 7.**
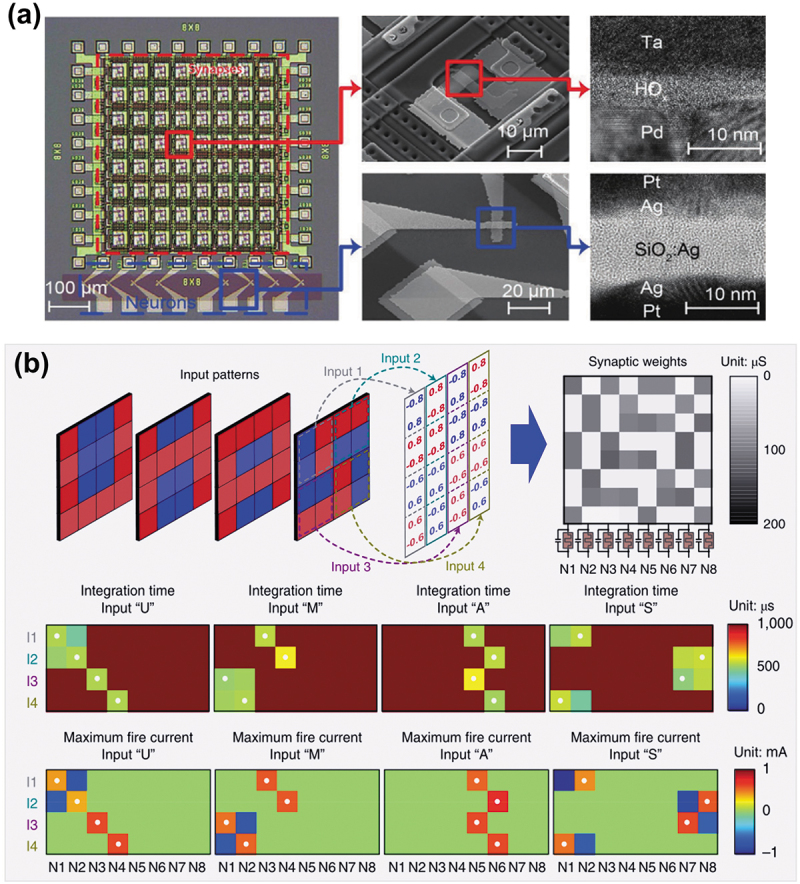
Hardware implementation of SNN computing based on memristive spiking neurons. (a) Optical micrograph of an integrated full memristive neural network consisting of an 8 × 8 ITIR memristive synapse crossbar and eight memristive spiking neurons. (b) Fully integrated memristive neural network for pattern classification of four letters ‘UMAS’. Reprinted with permission from [83]. Copyright 2018 Springer Nature.

From a computational complexity perspective, the LIF model is one of the most widely used models in SNNs hardware [16,126–128]. Meanwhile, with the favorable size and power scaling of memristive device, there is a path toward an all-memristor neuromorphic SNN computing based on artificial LIF neurons [83,100,129]. For example, Wang et al. developed a fully memristive SNN with an unsupervised learning capability by integrating one-transistor-one-memristor (1T1R) memristive synapse crossbar and memristive artificial neurons in the same hardware system [83]. The Pt/SiO_x_N_y_:Ag/Pt volatile memristor can serve as an artificial LIF neuron, and a Pd/HfO_x_/Ta nonvolatile memristor in series with an n-type enhancement-mode transistor (1T1R) plays the role of synapse. An example of integrated chips comprising an 8 × 8 1T1R synaptic crossbar and eight memristive spiking neurons is shown in Figure 7(a). Based on this hardware system, the artificial memristive neurons can utilize the LIF function to enable unsupervised synaptic weight updating and pattern classification on fully memristive neural network (Figure 7(b)). Given that two-terminal memristor devices are scalable and stackable, this integrated fully memristive SNN could lead to future ultra-large-scale networks.

In SNN’s data encoding schemes, a spatiotemporal-coding scheme is more powerful, since it can encode the same information with fewer spikes, which improves the information density and energy efficiency [123,130]. Inspired by this scheme, Zhang et al. firstly proposed a hardware implementation of fully memristive SNN with temporal coding (TC) using novel LIF neuron circuit based on a NbO_x_ volatile TS memristor [131]. A rate coding (RC) neuron was first constructed, then over which a D-flipflop and a transfer gate were introduced to form the neuron circuit. The neuron could encode the different input intensity (synaptic current) into spiking latency and fire at most once within an inference window. The research workers further experimentally demonstrated a fully memristive TC SNN (256 × 5) for face recognition with high inference accuracy. Attributing to the one-spike scheme, the TC SNN features a better performance (20.1 TMACS/W under a 10 ns time-step) than the RC SNNs in terms of the inference speed, energy consumption, and lifetime of neurons.

### 5.2. Memristive dendritic neurons for SNN computing

**Figure 8.**
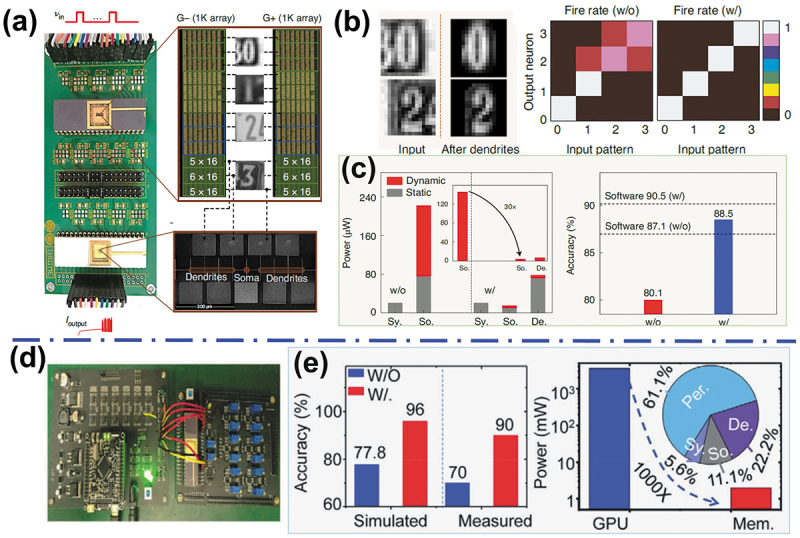
Hardware implementation of SNN computing based on memristive dendritic neurons. (a) Photograph of the neural network with memristive soma, dendrites, and synapse crossbar. (b) Dendritic neural network computing for image processing; comparisons of the neuron firing rates with and without artificial dendrites. (c) Comparison of the power consumption (left) and recognition accuracies (right) of this hardware system with/without dendrites. Reprinted with permission from [135]. Copyright 2020 Springer Nature. (d) Optical image of the designed dendritic neural network hardware platform on a printed circuit board. (e) Left: accuracy comparison for human motions recognition with/without dendritic functions; right: power consumption comparison for human motions recognition with dendritic functions running on GPU and memristors-based platform. Reprinted with permission from [136]. Copyright 2022 WILEY-VCH.

On the SNNs hardware discussed above, the artificial neuron is based on LIF function of biological neuron, without rendering more complex biodynamic behaviors. Actually, a complete biological neuron is composed of dendrites, a soma and an axon. The soma provides spiking integration and firing functions, while dendrites (branches of neurons that transmit signals between synapses and soma) play crucial hierarchical information-processing roles in biological neural networks, such as integrating postsynaptic signals nonlinearly and filtering out insignificant background information [132–134]. Li et al. experimentally demonstrated a fully integrated neural network with dendritic nonlinear integration and filtering functions implemented using three key computing components-artificial synapse, dendrite, and spike-firing soma. In the fully integrated memristive neural network, two 2 kilobyte (kB) HfO_x_-based non-volatile memristors arrays with 1T1R configuration serve as the artificial synapses, 32 TaO_x_/AlO_δ_-based dynamic memristors serve as the artificial dendrites and 3 NbO_x_-based Mott memristors serve as the artificial somas (Figure 8(a)) [135]. This network, equipped with functional dendrites, not only filtered background noise but also amplified the key signals in the image (Figure 8(b)). Compared with a neural network without dendrites, dendritic neural network’s test results have exhibited significantly reduced power consumption with improved recognition accuracy when performing a digit recognition task (Figure 8(c)). Based on this bio-plausible dendritic neuron, the team has also developed recently a similar dendritic neural network by integrating the dendritic neuron with memristor-based artificial synapse arrays (Figure 8(d)), and realized the Nanyang Technological University-Red Green Blue (NTU-RGB) human motion dataset recognition [136]. This scheme showed about 20% improvement in accuracy for the fully memristive hardware with dendrites, and 1000× power efficiency advantage compared to the graphics processing unit (GPU) (Figure 8(e)). The more complete memristive neuron with dendritic function is one of key building block for implementing more bio-plausible SNN that can handle complex spatial-temporal tasks with high accuracy and low power.

## 6. Memristive neuron for neuromorphic sensing

Beyond neuromorphic computing application, memristive neurons have also been used to construct human-like interactive neuromorphic sensing computing system. By using perception computing system based on artificial memristive neurons, continuous analog sensing signals from the environment can be converted into discrete spike signals without using conversion circuits [29,137,138]. Compared with traditional perception system, this bio-inspired perception system has simpler circuits, faster processing speed, lower energy consumption and cost, and endows the system with adaptive, perceptual fusion, and other biological characteristics. Construction of such perception system hardware would require devices in which neuronal information processing capability will be coupled with various sensing functionalities (e.g. tactile, visual, olfactory, etc.). In this section, we focus on the recent developments for bio-inspired interactive neuromorphic sensing system-based memristive neurons and their biomimetic applications.

### 6.1. Artificial haptic sensory system

**Figure 9.**
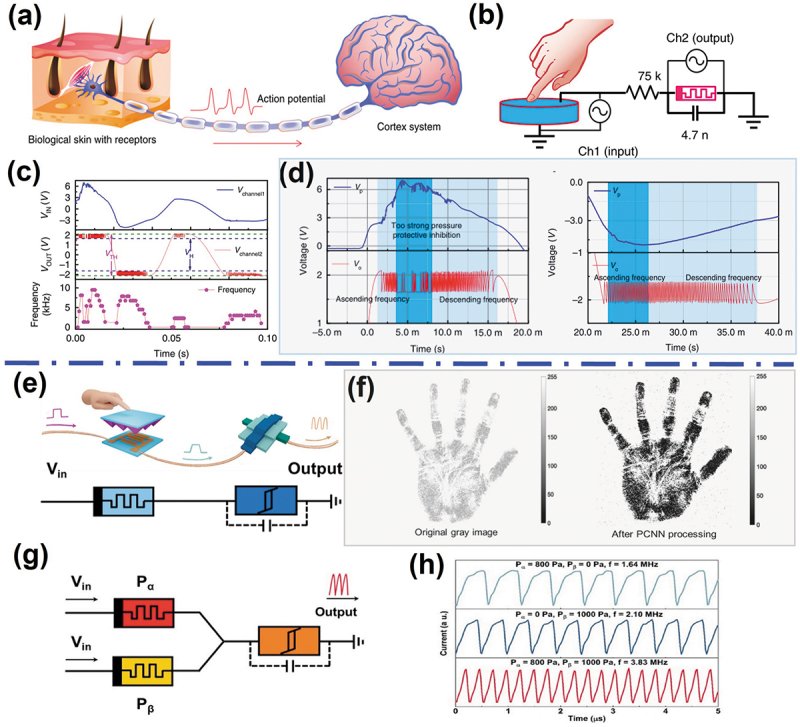
Artificial haptic sensory system based on memristive neurons. (a) Schematic of a biological haptic sensory system. (b) Schematic illustration of the power-free artificial spiking mechanoreceptor system based on memristive spiking neuron. Here, the tactile sensory and voltage signal are generated by the piezoelectric device. (c, d) The output signals of the artificial spiking sensory system under different pressure intensities. Reprinted with permission from [29]. Copyright 2020 Springer Nature. (e) Schematic illustration and circuit diagram of the artificial mechanical sensory system composed of a pressure sensor (left) and a NbO_x_-based mott memristor (right). (f) Pulse coupled neural network for tactile information sensation enhancement based on the artificial mechanoreceptor. (g) Bio-inspired tactile integration can be successfully implemented by two parallel pressure sensors (P_α_ and P_β_) and a series memristor. (h) Electrical spiking frequency enhanced when external stimuli were applied on both sensors. Reprinted with permission from [139]. Copyright 2021 American Chemical Society.

Inspired by the human skin, which is capable of perceiving external pressure stimuli, and transduce stimuli to the brain via the nervous system to form haptic perception (Figure 9(a)) [140–142]. By imitating biological action potentials, signal transmission, and neural interfaces in sensory process, an intelligent interactive system endowed with an integrated sensory neuronal loop can be achieved. In bio-inspired tactile perception system, tactile sensors are connected to artificial memristive devices. Based on volatile TS properties and internal dynamic behaviors of memristors, the output signals of the perception system will vary with degrees of external stimuli. Zhang et al. developed a novel bio-inspired spiking afferent nerve based on a specifically designed Mott memristor [29]. As shown in Figure 9(b), the artificial spiking somatosensory system, consisting of a mechanical sensor, a series resistor and an artificial spiking afferent nerve with a NbO_x_-based volatile TS memristor, can transform input pressure information (analog signals) into correlated output action potentials (spiking frequency signals). For such the somatosensory system, the input stimuli were related with the voltage generated by mechanoreceptors, and the oscillation frequency was related to the spiking frequency of the neuron, which in turn depends on the input intensity. Interestingly, this perception system had a self-powered device (piezoelectric device), the input voltage signal was generated by the change of a piezoelectric device. This system shows a quasi-linear relationship between input intensity and spiking frequencies under proper stimuli, and tends to reduce firing frequency when noxious stimuli are provided, which faithfully emulates the haptic perception nervous system. The dynamic frequency response can be clearly observed under different pressures, which has the same trend with the generated voltage (Figure 9(c)). Notably, when the pressure is too high, that is noxious stimuli, a high peak voltage is generated, which makes the system stop firing spiking (protective inhibition), as shown in Figure 9(d). The protection mechanism is similar to the self-protective behavior of the human perceptual nervous system [143,144].

Moreover, artificial biological efferent nerve has been further applied, Li et al. reported a skin-inspired artificial mechanoreceptor by integrating a highly sensitive resistive pressure sensor with a NbO_x_-based volatile TS memristor, to emulate the tactile sensation and perception in natural skin, respectively (Figure 9(e)) [139]. The stimuli intensity-dependent spiking in biological nervous system was realized by converting the external mechanical stimuli into strength-modulated electrical spikes. Tactile sensation enhancement was further implemented to render tactile perception more recognizable with the pulse coupled neural network (Figure 9(f)). In addition, the artificial mechanoreceptor could integrate coding signals from parallel tactile sensors, and encode them into unified electrical spikes, providing faster tactile neural processing (Figure 9(g,h)).

### 6.2. Artificial visual sensory system

**Figure 10.**
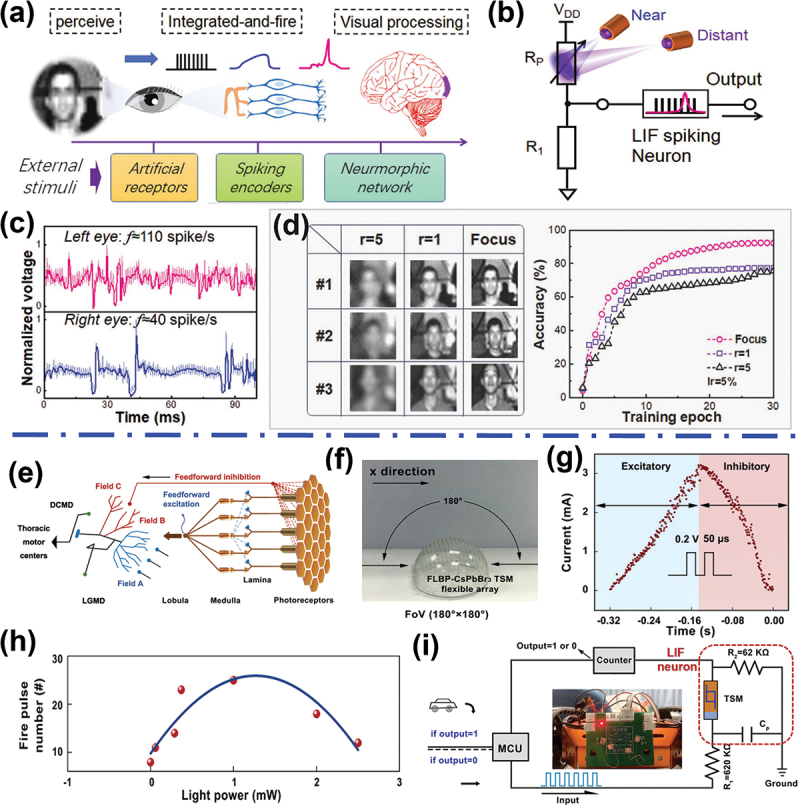
Artificial visual sensory system based on memristive neurons. (a) Schematic of the human visual sensory system. (b) The circuit scheme for the visual sensory system based on photoelectric memristive spiking neuron. (c) The firing rate of the left eye and the right eye at a certain distance, respectively. (d) A spiking visual neural network for image recognition based on the artificial binocular visual system. Reprinted with permission from [147]. Copyright 2022 WILEY-VCH. (e) Schematic diagram of the anatomical organization of the visual system with the LGMD neuron. (f) Photograph of the device-level the hemispherical shaped biomimetic compound eye based on flexible Ag/FLBP-CsPbBr_3_/ITO-based memristor crossbar, where FLBP stands for few-layer black phosphorous nanosheets and ITO is indium tin oxide. (g) Excitatory and inhibitory response of the memristor to a looming light stimulus with simultaneously applied electric pulses. (h) The artificial LGMD neuron fire pulse number vs. light power. (i) Schematic diagram illustrating the robot car’s decision-making with optical signal processing ability based on memristive spiking neuron. Reprinted with permission from [148]. Copyright 2021 Springer Nature.

With the rapid development of future advanced AI robotic systems, there is urgently needed to develop superior and intelligent artificial visual perception neural systems for diverse intelligent scenarios (such as machine vision, security monitoring, driverless cars and military defense) [9,145]. Vision is remarkable in that it empowers us to detect things as tiny and close as a mosquito on the tip of the nose, or immense and far away as a galaxy near the edge of the universe [146]. The mammalian visual perception system begins with the eye, and can obtain nearly 80% of the information received from the external world. At the back of the eye is the retina, which can contain photoreceptors specialized to convert light information into neural activity, and deliver to the brain for visual processing (Figure 10(a)). The human retina also can eliminate redundant and useless visual input processing and dramatically accelerates the extraction and detection of motion target features [149]. Therefore, building visual perception systems capable of sensing, converting, transmitting, and recognizing is of significant implication for future electronics, whereas a photoelectric memristive spiking neuron with high biological plausibility is the prerequisite.

Recently, Chen et al. presented a highly bio-realistic visual sensory system based on photoelectric spiking neurons for visual depth perception [147]. As illustrated in Figure 10(b), the perception system was built based on LIF spiking neuron, a photoresistor (R_p_) and a resistor (R_1_). The firing spikes of the perception spiking neuron generated by the TaO_x_-based memristive neuron (i.e. LIF neuron) have a biological plausibility with frequency range of 1–200 Hz and the sub-microwatts power consumption. Interestingly, an artificial binocular visual system with two sensory neurons could emulate firing rate difference between left and right eyes, which could be used to infer the depth of the vision (Figure 10(c)). The artificial binocular visual system can take advantage of the depth perception to improve the accuracy of a hardware neural network’s image recognition (Figure 10(d)). Another type of implementation is based on two memristors, one of which can be motivated by optical signals and the other is responsible for signal processing. Pei et al. reported a fully memristor-based artificial visual perception nervous system, and a quantum-dot-based photoelectric memristor can receive and respond to external optical stimuli [150]. This photoelectric memristor and a nanosheet-based memristor construct the artificial visual system and implement LIF neuron functions. In an automobile meeting scenario, the self-regulation process of a speed meeting control system in driverless automobiles can be accurately and conceptually implemented by this system.

Inspired by the obstacle avoidance during locust movement, the lobula giant movement detector (LGDM) can rapidly respond to fast-moving or small objects and efficiently triggers escape behavior (Figure 10(e)). The principle derived from LGMD is ideal for designing artificial vision systems, which can inform intelligent robots of the impending collision [151,152]. Recently, Wang et al. reported a biomimetic compound eye based on artificial LGMD visual neuron by using flexible light-mediated Ag/FLBP-CsPbBr_3_/ITO-based memristor crossbar [148]. The flexible memristor crossbar with larger acceptance angles than individual devices, which can distinguish the direction and velocity of approaching objects (Figure 10(f)). When the continuous pulsed light is applied to the TS device, the conductance of the device: first increases, then peaks, finally decreases, which is similar to the excitatory and inhibitory response of LGMD neuron toward the looming object (Figure 10(g)). The multi-process characteristic of the light-modulated is attributed to the temporal heat summation dynamic effect. The moderate Joule heating effect will accelerate Ag drifts process to induce the formation of Ag-conductive-filaments in the device, resulting in the excitatory behavior of the artificial LGMD neuron; while the considerable Joule heating effect will induce the rupture of Ag-conductive-filaments, resulting in the inhibitory behavior of the artificial LGMD neuron. Given the optical modulated non-monotonic behavior of the TS device, the artificial LGMD neuron could be built by the simple LIF neuron circuit. As shown in Figure 10(h), the LGMD neuron can respond to optical stimulus and encode them into spikes with the non-monotonic response. The artificial LGMD neuron was further integrated with circuits to implement the collision avoidance function of smart robots with wide field-of-view detection (Figure 10(i)).

### 6.3. Artificial multisensory system

**Figure 11.**
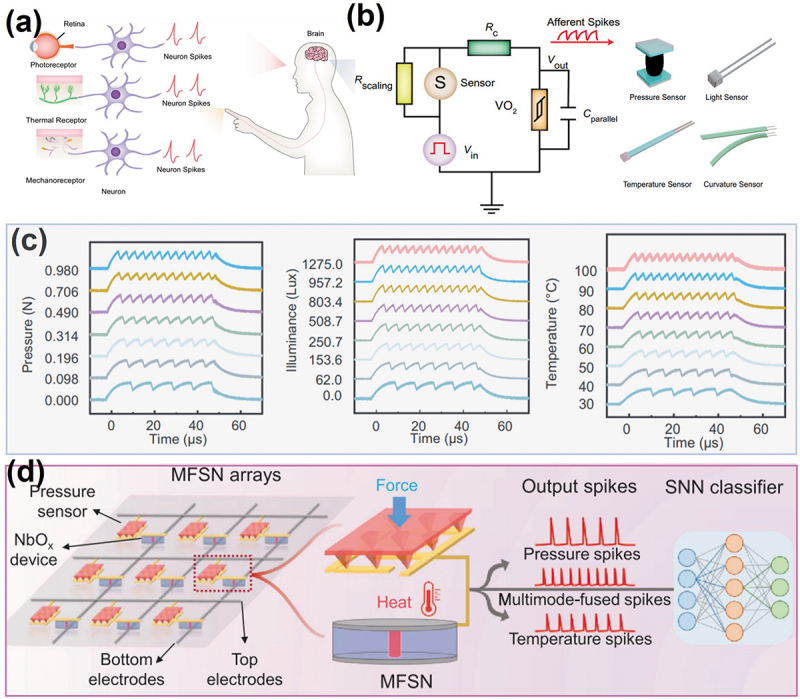
Artificial multisensory system based on memristive neurons. (a) Illustration of human multisensory biological system. (b) Schematic of spike-based artificial neuromorphic sensory system. The calibratable artificial sensory neuron combined with various sensors (pressure sensor, temperature sensor, light sensor, and curvature sensor). (c) Response of artificial spiking sensory neuron to different stimuli (pressure, light intensity, and temperature). Reprinted with permission from [153]. Copyright 2022 Springer Nature. (d) Schematic of the artificial multisensory neuromorphic computing system consisted of a multimode-fused spiking neuron (MFSN) array and an SNN classifier. The MFSN cell is composed of a NbO_x_-based mott memristor and a pressure sensor. Reprinted with permission from [137]. Copyright 2022 WILEY-VCH.

As mentioned above, the bio-inspired interactive neuromorphic perception system handled by single-perception physical signal has demonstrated remarkable progress. The world is multimodal, and the human multisensory nervous system enables people to learn and adapt to external environment by integrating different sensory information, such as visual, tactile, auditory, olfactory and taste (Figure 11(a)). Making intelligent robotic sensing more human-like requires a bio-inspired multimodal perception system with high-level cognitive sensing and processing of multimodal environmental information.

To handle multimode physical signals, some researchers proposed multi-perception neuromorphic computing system based on emerging memristive device [154–156]. Recently, Yuan et al. designed a spike-based artificial multisensory system consisting of calibratable artificial sensory neurons (CASN) based on the Mott memristor with excellent uniformity [153]. As shown in Figure 11(b), the CASN comprises of four core components: a sensor, a VO_2_-based Mott memristor, a parallel capacitor, a series resistance and a scaling resistance. Various sensory neurons can be implemented based on the CASN by changing sensor types (pressure, curvature, illuminance and temperature sensor). The scaling resistor can be further used to accommodate varied types of sensors by adjusting their various resistance ranges to the desired regime. Based on this principle, a multi-perception system capable of encoding different sensory information into spikes is demonstrated experimentally (Figure 11(c)). Therefore, this memristive multisensory system provides a potential as an interface between the external environment and neuromorphic computing systems. A neuromorphic perceptual computing system was implemented for mixed national institute of standards and technology (MNIST)-based pressure image classification. Simulation results show that combination of the spiking pressure sensory neurons with SNN can successfully classify patterns of different pressure images with >90.33% classification accuracy after 50 training epochs. However, in this artificial multi-perception system, the implementation of multiple sensory requires changing the sensor in the neuronal circuit without the process of sensory fusion, which is still a certain gap with human multimodal perception system. To solve this problem, the neuromorphic perception systems need to be improved based on further study of human perception mechanism. Zhu et al. proposed a multimode-fused spiking neuronal system to achieve human-like multisensory function (Figure 11(d)) [137]. This system heterogeneously integrated a pressure sensor and a NbO_x_-based Mott memristor with thermal response characteristics. When applying both pressure and temperature stimuli to the system, the two sensory inputs can be encoded into one spike train, showing its superior data compression and conversion capabilities. Two sensory signals are distinguished from fused spikes by decoupling the output frequencies and amplitudes, supporting multimodal tactile perception. Unfortunately, this system only supports tactile perception (i.e. temperature and pressure), and a bio-inspired interactive multisensory-fused still need to be further developed.

## 7. Challenges and outlooks

So far, we have comprehensively reviewed recent progress and breakthrough in memristive neurons, along with their potential applications in neuromorphic sensing and computing. From the perspective of biological neuron, various neuron models, including IF, LIF and HH neurons, are briefly discussed. This is followed by specific discussions on the internal mechanisms that endow memristive devices with rich resistive switching dynamics to emulate the functions of biological neurons. Then, various memristive neurons with rich spiking behaviors have been discussed. In addition, we introduce the recent development on SNNs hardware systems based on memristive neurons. Finally, we have discussed memristive neuron working for interactive neuromorphic perception systems in hardware. Although memristive neurons promote fast progress for high-efficiency intelligent sensing and computing, they are still at the infant stage and facing some challenges.

From the device level, the requirements for memristive devices to realize neural functions are: volatile switching behavior, low threshold voltage, low cycle-to-cycle (C2C) and device-to-device (D2D) variations, high thermal stability, and low power consumption [35,157]. While the randomness of the device might be beneficial to the probabilistic computing, it can also limit the learning ability of the network if the cells are too stochastic. In fact, the inherent stochastic of memristive device is mainly associated with the random formation/rupture of the conductive filaments in operation processes. The resistive materials can greatly affect the C2C stability. Especially in conductive filament devices, such as ECM devices, the overgrowth of conductive filaments and the random diffusion of conductive filament atoms will affect the device stability. To better control conductive filament, researchers proposed some approaches, such as selecting appropriate electrode materials, adding interface layer and doping the electrode or the dielectric layer [25,158,159]. These methods help to narrow down the variability of devices. Moreover, the CMOS compatible materials should be first considered for building memristive devices, as these materials are intensively investigated and the fabrication processes are highly feasible.

From the circuit level, memristive neuron circuits are desired to effectively emulate the firing behaviors. Some basic neuronal functions, such as leaky-integration-firing and refractory period, have been mimicked by simple neuronal circuits. Compared to biological neurons, the functions of the artificial neuron are still limited. Despite the artificial HH neuron circuit has implemented many advanced functions (Section 3.2), this circuit is complex, which is not suitable for large scale integration. Interestingly, a higher order memristor can enable rich neuron spiking dynamics by carefully tunning material composition and device structure. These neuronal functions occur only within a narrow subset of material compositions that support NDR, which is predicted by Chua’s theory of local activity. Present-day memristors are single first-order memristors that lack these complex dynamic properties. Using the high-order memristor to realize the rich neuronal functions will also be an interesting research topic. Moreover, memristive devices need to be equipped with transistor-based selectors or rectifying capabilities to realize a large-scale array [160]. The reported neuron array was still limited to very small scale till now, and lack of reliability to replace traditional CMOS-circuit-based neurons. How to further enhance the scale and stability of neuron circuits to the neural network hardware and realize proper training is the current challenge.

At the system level, many issues need to be solved before implementing neuromorphic computing and sensing based on memristive devices. In terms of neuromorphic computing system, extreme attention should be paid to the connections among a vast number of memristive synapses and neurons in a fully memristive neural network. Till now, artificial neuron devices have been studied separately from synaptic devices, and there is a concern that they will not work with synaptic devices in a physical neuromorphic computing system. Although researchers have reported fully memristive neural networks (Section 5) recently, they focused on very small synaptic arrays and individual artificial neuron integration, which is still far from the complexity and computing capacity of the brain. In contrast to conventional CMOS technology, a novel non-CMOS approach (i.e. nanowire networks) provides a complex structure for the network circuit, and embeds the higher interconnection of the resistance switch memory junction [161–163]. This self-assembled network of memristive elements would give a novel idea to solve the interconnection of a vast number of synapses and neurons. Memristive SNNs are still facing challenges in learning algorithms. The well-established back-propagation and stochastic gradient descent have shown impressive performance in the field of ANNs. However, these learning algorithms do not map directly to SNNs, because spiking neurons do not have differentiable activation functions in SNNs [164,165]. Thus, it is of utmost importance to develop spike-based efficient training algorithms for SNNs.

In terms of neuromorphic-sensing system, how to match the varied types of sensors with the memristive neuron is a huge challenge. A scaling resistor has been incorporated to configurate the neuronal circuit (see Section 6). However, this scheme only focuses on converting perceptual signals into spikes, sacrificing the performance of the sensor system. Therefore, it is needed to evaluate and compare the sensor characteristics, specifically the sensitivity, selectivity, and reliability, in artificial sensory neurons, to further realize highly matching of signals between biology and electronics. In addition, mechanical compliance of artificial sensory system is important for the biocompatible neuromorphic electronics. The artificial sensory neuron should be tolerant of the mechanical deformation. The structures and materials of flexible device need to be optimized to achieve stable neuronal responses based on various sensory signals regardless of mechanical strain. In the future, a stretchable and biocompatible artificial neuron will expand to skin-attachable and implantable neuromorphic electronics for wearable computing, health monitoring, and sensorimotor neural signal transmission.

In summary, as the memristive neuron described in this review is just a very early form of an artificial dynamic neuron, it is still not comparable to the sophisticated human brain but has the intriguing potential at the same time. The memristive neurons should be integrated in the form of an array and combined with synaptic arrays to achieve energy-efficient system-level neuromorphic computing in the future. Meanwhile, the artificial sensory neuron chip should be appropriately designed and integrated with SNN computing chip, to realize human-like integrated interactive sense-memory-computation. It is noteworthy that the development of bio-inspired interactive neuromorphic systems is a highly interdisciplinary task. Collaborations among neuroscientists, material scientists, device engineers, and system-level engineers are indispensable for developing artificial neuromorphic hardware that have more biomimetic functions. Advanced interactive neuromorphic electronic systems based on memristive devices are expected to appear soon, and will revolutionize the future of intelligent perception-computing and smart robotics.
